# Association of ABO Blood Groups and Obesity in Patients With Diabetes Mellitus in King Abdulaziz University Hospital

**DOI:** 10.7759/cureus.51569

**Published:** 2024-01-03

**Authors:** Reem Mohammed Alqahtani, Sohaib Essam Althagafi, Aseel Ahmed Althagafi, Jalal Mohammed Alsayyad, Awatif Abdualaziz Saeedi, Obadah Suhail Mishiming, Khaled A Yaghmour, Mohammed Anwar Jan

**Affiliations:** 1 Department of Family Medicine, Faculty of Medicine, King Abdulaziz University Hospital, Jeddah, SAU; 2 Faculty of Medicine, King Abdulaziz University, Jeddah, SAU; 3 Department of Family Medicine, Faculty of Medicine, King Abdulaziz University, Jeddah, SAU

**Keywords:** overweight, blood type, diabetes mellitus, blood group, obesity

## Abstract

Background: Differences in the blood, resulting from the presence or absence of antigens corresponding to specific blood types, have indirect implications for susceptibility to diseases. The aim of this study was to examine the association between the ABO type and obesity in patients with diabetes mellitus.

Methods: This is a cross-sectional observational study that was conducted at King Abdulaziz University Hospital (KAUH) in Jeddah, Saudi Arabia. Data were collected using a simple random method through hospital records during the period between August and September 2022. Patients with type 1 diabetes mellitus (T1DM) and T2DM were included in this study. Binary logistic regression analysis was used to identify the association between blood group and obesity.

Results: A total of 411 patients were involved in this study. More than half of the patients (54.3%; n= 223) are diagnosed with T1DM. O-positive was the most common blood group type among the patients, accounting for 38.6% (n= 161). Only 23.6% (n= 97) of the patients were classified as having normal weight. Around 32.6% (n= 134) of the patients were classified as having the pre-obesity stage. More than one-third of the patients (35.1%; n= 145) were classified as being obese. There was no statistically significant difference between the patients in terms of their blood type group and its association with obesity (p>0.05).

Conclusion: Many diabetics are overweight or obese, according to this study. This shows the importance of weight management for diabetes treatment. Many patients were overweight, emphasizing the importance of obesity prevention and diabetes care. Most patients were O-positive, according to blood type tests. Previous research suggests that blood types may be linked to diabetes. However, this study found no significant relationships. More research is needed to understand the complex link between blood types, weight, and diabetes.

## Introduction

ABO blood group antigens in humans are observed in different transfusion phenotypes and are glycoconjugate structures present on the surface of erythrocytes. These antigens have significant implications in cellular physiology and pathology [1]. The physiological importance of ABO antigens and the accompanying natural anti-A and anti-B isoagglutinin is not yet fully comprehended. However, their significance has been established in the contexts of blood transfusion as well as cell, tissue, and organ transplantation [2]. Differences in the blood, resulting from the presence or absence of antigens corresponding to specific blood types, have indirect implications for susceptibility to diseases [3,4]. Prior research has demonstrated significant correlations between ABO blood types and a range of medical conditions, encompassing cancer, cardiovascular disease, cognitive problems, and metabolic disorders, including hypertension, obesity, and diabetes mellitus [1,5,6]. Diabetes mellitus is a prevalent metabolic disorder characterized by impairments in insulin synthesis and/or function, and it ranks among the most prevalent diseases globally. This condition is closely linked to elevated rates of morbidity and mortality [7]. Around seven million of the Saudi population are diabetic, and almost three million are pre-diabetic [8]. The prevalence of DM is about 20-25% in Saudi Arabia [8]. The association between ABO and Rh blood group phenotypes and numerous illnesses, such as type 2 diabetes mellitus (T2DM), has been demonstrated [9]. This association may be attributed to inherited variations in immunoglobulins [9]. Several factors, such as genetic predisposition, immunological factors, and environmental factors, play a crucial role in the development and progression of T2DM. These factors have a substantial impact on the etiology and consequences of the disease. The ABO and Rh blood types exhibit genetic determination, suggesting a potential genetic association with other disorders that possess a significant genetic basis, such as DM [9]. Genes associated with susceptibility to T2DM are located inside the chromosome area 1q21-q23 in humans. Additionally, the ABO blood group genes, which often exhibit changes, are situated in the chromosome region 9q34.2 [10,11]. Enhanced understanding of the associations between T2DM, obesity, and the ABO or Rh blood groups could potentially contribute to the prevention of T2DM by enabling the identification and avoidance of potential predisposing factors. Additionally, this knowledge may aid medical professionals in identifying patients who are at a higher risk of developing this disease.

Recent research has indicated that certain ABO-type antigens possess a propensity to increase the likelihood of developing T2DM, while others exhibit protective properties against the disease. Furthermore, various studies have demonstrated that the presence or absence of Rh blood group antigens might serve as either protective or risk factors for the development of T2DM and other metabolic disorders [12-14]. Consequently, our objective was to examine the association between the ABO type and obesity in patients with diabetes mellitus.

## Materials and methods

Study design

This is a cross-sectional observational study that was conducted at King Abdulaziz University Hospital (KAUH) in Jeddah, Saudi Arabia. Data were collected using a simple random method through hospital records during the period between August and September 2022. The targeted study population was defined in the medical records system of the hospital, and then, a random sample of the medical records for patients who meet our inclusion criteria was taken. The sample was chosen based on random numbers that were generated using Excel.

King Abdulaziz University Hospital is considered the first teaching hospital in the Kingdom of Saudi Arabia and provides distinguished medical services in the field of ophthalmology, ear, nose, and throat medicine at the hands of experienced and competent consultants, doctors, and technicians using the best infrastructure and technical resources in the Kingdom. In addition to primary, secondary, and tertiary health care services in the field of ophthalmology, ear, nose, and throat, the hospital provides primary health care in the following specialties: internal medicine, surgery, pediatrics, obstetrics and gynecology, anesthesia, psychiatry, X-rays, medical rehabilitation, laboratory medicine, and dental services.

Study population

Patients with type 1 diabetes mellitus (T1DM) and T2DM were included in this study. There is no restriction on the age or gender. Patients with a blood glucose level of less than 6.1 mmol/l and who had never taken any medication for diabetes were excluded from the study. In addition, we excluded patients with drug-induced DM and gestational DM.

Clinical data

The clinical data of all patients who meet the inclusion criteria were extracted. Data were extracted from the medical records of the hospital. Extracted data included age, gender, BMI, and type of diabetes mellitus. The history for the following comorbidities was additionally taken into account: hypertension, malignancy, coronary heart diseases, congestive heart failure, cerebrovascular events, autoimmune disorders, thyroid diseases, chronic liver disease, rheumatological conditions, respiratory disease, and chronic renal failure.

BMI for the study participants was defined as per the World Health Organization classification [15]. An individual with a BMI exceeding 25 kg/cm^2^ is classified as overweight, whereas a BMI beyond 30 kg/cm^2^ is indicative of obesity. Besides, a BMI below 18.5 kg/cm^2^ was classified as underweight, 18.5-24.9 kg/cm^2^ classified as normal weight, and 25.0-29.9 kg/cm^2^ classified as pre-obesity [16].

Ethical approval

Ethical approval for this study was obtained from the Institutional Review Board at King Abdulaziz University Hospital, Jeddah, Saudi Arabia (Reference No: 460-23). Informed consent was obtained from all subjects involved in the study.

Statistical analysis

IBM SPSS Statistics for Windows, Version 29 (Released 2023; IBM Corp., Armonk, New York, United States) was utilized for data analysis. The normality of the data was checked using the histogram and normality measures (skewness and kurtosis). Mean and standard deviation were used to express normal distribution continuous variables and median and interquartile range for non-normally distributed data. Categorical data were presented using frequency and percentage. Binary logistic regression analysis was used to identify the association between the blood group and obesity. The p-value is set at <0.05 to indicate statistical significance.

## Results

Table 1 presents the baseline characteristics of the study participants. A total of 411 patients were involved in this study. The median age of the patients was 51.0 years (40-56.0). More than half of them (57.2%; n= 235) were males. Around 49.6% (n= 204) of the patients were Saudis. The mean BMI for the patients was 28.9 kg/cm^2^ (12.4). More than half of the patients (54.3%; n= 223) are diagnosed with T1DM. O-positive was the most common blood group type among the patients, accounting for 38.6% (n= 161).

**Table 1:** Participants' baseline characteristics

Variable	Frequency	Percentage
Median age (years) (interquartile range)	51.0 (40.0-56.0)	
Gender		
Male	235	57.2%
Nationality		
Saudi	204	49.6%
Body mass index (kg/cm^2^) (standard deviation)	28.9 (12.4))	
Type of diabetes mellitus		
Type 1 diabetes mellitus	223	54.3%
Type 2 diabetes mellitus	188	45.7%
Blood group type		
O+	161	38.6%
A+	128	31.4%
B+	61	15.0%
AB+	26	6.4%
AB- AB+AAASAS	11	2.7%
B-	10	2.5%
A-	7	1.7%
O-	7	1.7%

Obesity profile

Table 2 presents the obesity profile of the patients. Only 23.6% (n= 97) of the patients were classified as having normal weight. Around 32.6% (n= 97) of the patients were classified as having the pre-obesity stage. More than one-third of the patients (35.1%; n= 145) were classified as being obese.

**Table 2:** Participants' obesity profile

Variable	Frequency	Percentage
Body mass index category		
Underweight	35	8.7%
Normal weight	97	23.6%
Pre-obesity	134	32.6%
Obese	145	35.1%

Association between the blood group type and obesity status

Table 3 presents the findings of binary logistic regression analysis. Both unadjusted and adjusted regression models showed that there was no statistically significant difference between the patients in terms of their blood type group and its association with obesity (p>0.05).

**Table 3:** Binary logistic regression analysis * Adjusted regression model for age, gender, and type of diabetes.

Blood group type	Unadjusted odds ratio of being obese (95% confidence interval)	p-value	Adjusted odds ratio of being obese* (95% confidence interval)	p-value
A- (Reference group)	1.00		1.00	
A+	1.18 (0.22-6.33)	0.848	1.81 (0.31-10.66)	0.511
B-	2.50 (0.32-19.53)	0.382	1.13 (0.67-1.92)	0.643
B+	1.41 (0.25-7.88)	0.695	0.37 (0.10-1.40)	0.142
AB- AB+	0.25 (0.02-3.47)	0.301	0.99 (0.52-1.90)	0.986
AB+	1.56 (0.25-9.65)	0.631	6.26 (0.75-52.05)	0.089
O-	3.33 (0.36-30.70)	0.288	0.72 (0.28-1.82)	0.485
O+	1.24 (0.23-6.60)	0.802	0.37 (0.08-1.83)	0.224

## Discussion

This study sought to examine the relationship between ABO blood types and obesity in individuals diagnosed with diabetes mellitus. This study found that participants with diabetes mellitus had an average BMI of 28.9 kg/cm^2^, which classifies them as pre-obese according to the WHO classification [17]. Some studies have established a connection between higher BMI and increased blood glucose levels in diabetic patients [18,19]. However, other studies have not addressed this association with an increased risk of complications [20]. The reason behind this association is believed to be the excessive accumulation of body fat, which impairs the secretion of hormones responsible for transporting insulin into cells, leading to type 2 diabetes [19]. When diabetes is caused by obesity, addressing the issue of elevated BMI becomes crucial for effective treatment. Studies have shown that elevated levels of ectopic adipose tissue play a significant role in driving the development of type 2 diabetes [21].

This study revealed that 23.6% of the patients had a normal weight, while approximately 32.6% were classified as pre-obese. Furthermore, 35.1% of the patients were categorized as obese. These obesity findings differ from another study conducted in Saudi diabetic patients, where only 13.2% had a normal weight, 27.9% were overweight, and 57.8% were obese [22]. This indicates a high prevalence of obesity among diabetic patients. In a previous study examining the relationship between diabetes, body weight, and primary healthcare support in 49 developing countries, it was concluded that preventing abnormal body weight and improving healthcare support for diabetes are crucial steps in addressing the unfavorable diabetes epidemic in developing countries [23].

Among the study patients, the O-positive blood group was the most common, accounting for 38.6% of the total. In contrast to our findings, previous studies in Italy and Trinidad reported a higher prevalence of the B blood group among diabetic patients [24]. It was found that B blood group was more prevalent among diabetic patients [25]. It is believed that there is a connection between blood groups and type 2 diabetes due to shared genetic and immunologic factors [26]. However, other studies conducted in Bangladesh, India, Glasgow, and Germany have either found no association or no significant association between blood groups and type 2 diabetes [27-31]. On the contrary, a study conducted in Malaysia found that blood group B was significantly more common among patients with type 2 diabetes mellitus compared to our study [28]. It accounted for 35.71% of cases, whereas in the control group, it was only 22.52%. This pattern of blood group B being more prevalent among diabetes mellitus patients has also been observed in other studies [25,32]. It is worth noting that, in the Malaysia study, this difference compared to the control group was not statistically significant. However, their findings suggest a potential association between blood groups A and O and type 2 diabetes mellitus. Curiously, there seems to be a negative correlation between these blood groups and diabetes, suggesting that they are less common in people with the disease and may provide some level of protection against it [28].

Furthermore, it is worth noting that different blood types can have notable physiological variations among individuals [33,34]. Previous studies have suggested a potential connection between ABO blood groups and overweight and obesity [35,36]. However, our study findings indicate that there is no statistically significant difference between patients in terms of their blood type groups and their association with obesity. In contrast, another study revealed that different blood groups are linked to varying levels of obesity. Specifically, females with blood type O+ exhibited the highest level of obesity, while males with blood types O- and AB- demonstrated the lowest levels of obesity [33]. In contrast, numerous other studies have reported similar findings to ours, showing no significant statistical disparities between ABO blood group and conditions such as overweight, obesity, or increased BMI [37,38]. It is worth noting that overweight and obesity are common among the population in Saudi Arabia [39]. Various factors, including age, gender, education level, exercise habits, and co-existing medical conditions, have been linked to higher BMI in the Saudi population [40]. However, a study conducted specifically among the Saudi population found that individuals with blood group O had the highest BMI [37]. Notably, there were no noteworthy disparities detected in BMI, Rh factor, or sex across different ABO blood groups. In addition, their study yielded similar results to ours, indicating that there is no statistically significant relationship between Rh or blood type and BMI [37].

Furthermore, the research demonstrates that a significant number of individuals with diabetes are afflicted with excessive body weight or obesity, underscoring the critical necessity of weight management for the purpose of achieving efficient treatment of diabetes. A substantial proportion of patients were identified as obese, underscoring the significance of preventing excessive weight and enhancing diabetes assistance. The study also examined blood groups and determined that O-positive was the predominant blood type among patients. Previous studies suggested potential associations between blood groups and diabetes. However, this study did not uncover definitive correlations. The intricate nature of the relationship between weight, blood groups, and diabetes needs further research to achieve a thorough knowledge.

Study limitations

There are constraints to this study. This investigation was conducted at a single location. This could potentially impact the applicability of our study results. The utilization of a cross-sectional study design limited our capacity to establish a causal relationship between the variables under investigation. Hence, it is imperative to exercise caution when interpreting our findings.

## Conclusions

A significant number of individuals with diabetes are overweight or obese, according to this study. A significant proportion of the patients were found to be overweight, highlighting the criticality of preventing obesity and enhancing diabetes care. Previous research has indicated the possibility of associations between blood groups and diabetes. Nevertheless, this research failed to identify any significant association between the blood type and obesity. Due to the complex nature of the relationship between blood groups, weight, and diabetes, additional research is required to fully comprehend it.
